# A potentially crucial role of the *PKD1* C-terminal tail in renal prognosis

**DOI:** 10.1007/s10157-017-1477-7

**Published:** 2017-10-05

**Authors:** Eiji Higashihara, Shigeo Horie, Moritoshi Kinoshita, Peter C. Harris, Takatsugu Okegawa, Mitsuhiro Tanbo, Hidehiko Hara, Tsuyoshi Yamaguchi, Kaori Shigemori, Haruna Kawano, Isao Miyazaki, Shinya Kaname, Kikuo Nutahara

**Affiliations:** 1 Department of ADPKD Research, Kyorin University School of Medicine, 6-20-2 Shinkawa, Mitaka, Tokyo 181-8611 Japan; 2 Department of Urology, Kyorin University School of Medicine, Tokyo, Japan; 3 Department of Radiology, Kyorin University School of Medicine, Tokyo, Japan; 4 Department of Nephrology, Kyorin University School of Medicine, Tokyo, Japan; 5 Department of Urology, Juntendo University School of Medicine, Tokyo, Japan; 6 Diagnostic Division, Development Department, Otsuka Pharmaceutical Co., Ltd, Tokushima, Japan; 7 Division of Nephrology and Hypertension, Mayo Clinic, Rochester, MN USA

**Keywords:** Autosomal dominant polycystic kidney disease (ADPKD), Genotype/phenotype correlation, *PKD*1 mutation, Renal survival

## Abstract

**Background:**

Autosomal dominant polycystic disease (ADPKD) often results in renal failure. Recently, allelic influences of *PKD1* mutation types on renal survival were extensively investigated. Here, we analyzed integrated influences of *PKD1* mutation types and positions on renal survival.

**Methods:**

We included 338 (82 pedigrees) and 72 (12 pedigrees) patients with *PKD1* and *PKD2* mutations, respectively, identified through comprehensive gene analysis of 101 probands with ADPKD. Genetic testing was performed using next-generation sequencing, long-range PCR, and multiplex ligation-dependent probe amplification. Pathogenic mutations were identified by a software package-integrated seven databases and provided access to five cloud-based computing systems.

**Results:**

Mean renal survivals of carriers with *PKD1* non-truncating-type mutations at positions upstream of G-protein-coupled receptor proteolytic site (GPS-upstream domain), transmembrane domain, or cytoplasmic C-terminal tail (CTT) domain were 70.2, 67.0, and 50.1 years, respectively (*P* < 0.0001); renal survival was shorter for mutation positions closer to CTT domain, suggesting its crucial role in renal prognosis. Furthermore, in truncating-type mutations, strong inactivation is anticipated on nucleotides downstream from the mutation site, implying CTT domain inactivation irrespective of mutation site. Shorter mean renal survival was found for *PKD1* truncating-type than non-truncating-type mutation carriers (*P* = 0.0348); mean renal survival was not different between *PKD1* 3′- and 5′-region truncating-type mutation carriers (*P* = 0.4375), but was shorter in *PKD1* 3′-region than in 5′-region non-truncating-type mutation carriers (*P* = 0.0014). Variable strength of CTT domain inactivation might account for these results.

**Conclusions:**

Aforementioned findings indicate that CTT domain’s crucial role in renal prognosis needs further investigation by larger studies **(**ClinicalTrials.gov; NCT02322385).

**Electronic supplementary material:**

The online version of this article (doi:10.1007/s10157-017-1477-7) contains supplementary material, which is available to authorized users.

## Introduction

Autosomal dominant polycystic kidney disease (ADPKD) is the most common genetic kidney disease that leads to end-stage renal disease (ESRD) by the age of 60–70 years in approximately 50% of patients [1–4]. Approximately 85 and 15% of patients develop ADPKD due to *PKD1* and *PKD2* mutations, respectively [5–10]. Accumulating evidence indicates that these genes represent the most powerful determinant of disease severity in patients with ADPKD; survival to ESRD is 15–20 years less in patients with *PKD1*, than in those with *PKD2* mutations [8, 11].

*PKD1* mutations have significant allelic influences on renal phenotypic expression. The influence of truncating-type *PKD1* mutations was reported to be more severe than that of non-truncating-type *PKD1* mutations in renal survival studies [8, 12], and in an estimated glomerular filtration rate (eGFR) study [10]. Allelic influences of *PKD1* mutations on height-adjusted total kidney volume (htTKV) were observed only after the in silico division of non-truncating-type mutations [10]. The position of *PKD1* mutations (in the 5′- versus 3′-region) has been suggested to correlate with a renal phenotype including earlier mutations, resulting in an increased severity of the disease [13]. However, the effect of the position of *PKD1* mutations has not been reported in other large studies [8, 10].

*PKD1* encodes polycystin-1 (PC1), a 460-kDa protein with a large extracellular N-terminal region, a transmembrane region, and a cytoplasmic C-terminal tail (CTT) [14, 15]. *PKD2* encodes polycystin-2 (PC2), a member of the transient receptor potential family of non-selective cation channels. PC1 and PC2 interact through their CTT domains, and colocalize to the primary cilium, where they may perform mechanosensory functions [16]. The ciliary trafficking of PC1, and the formation of the PC1–PC2 complex, are regulated by the PC1–PC2 interaction [17, 18]. PC1 undergoes cleavage at its G-protein-coupled receptor proteolytic site (GPS), that is probably essential for its full function [19]. The CTT region of PC1 may also undergo cleavage, with the resulting CTT being translocated to the nucleus, where it initiates signaling [20, 21].

Few genotype/phenotype correlation studies have focused on the structure of PC1. Therefore, the results of the present study might contribute to our understanding of domain influences on *PKD1* mutations.

## Materials and methods

### Study design

Integrated allelic influences of the position and type of *PKD1* mutations on renal survival were examined in 410 patients with documented *PKD1* or *PKD2* mutations.

### Participants

The study recruited 101 probands with ADPKD (age >20 years) who visited the Kyorin University Hospital (KUH; *N* = 82) and the Juntendo University Hospital (JUH; *N* = 19) in Tokyo, between January 2014 and October 2015. ADPKD was diagnosed using previously described criteria [22]. Basic data for survival analysis were collected from pedigree members. The onset of ESRD was defined as the initiation of renal replacement therapy (RRT). The non-ADPKD subjects (control) included in this study were second-degree relatives of patients with ADPKD.

Informed consent was obtained from each participant. The study protocol adhered to the Declaration of Helsinki, and was approved by the institutional review boards of KUH and JUN (nos. 579 and 14-043, respectively). This study is registered in ClinicalTrials.gov, under accession no. NCT02322385.

### Mutation analysis

The detection and pathogenicity prediction of missense mutations were performed using next-generation sequencing (NGS), and long-range polymerase chain reaction (LR-PCR); data analysis was performed using software packages, as previously described [23]. Multiplex ligation-dependent probe amplification (MLPA) was used to analyze large genomic rearrangements, if pathogenic mutations were not detected by NGS.

PC1 and PC2 structural domains were classified using the Universal Protein Resource (UniProt) annotation [24] of UCSC, and a published study [25].

### Classification of *PKD1* mutation types and mutation strength groups

*PKD* mutations were classified into truncating- and non-truncating-type mutations. Truncating-type mutations included frameshift mutations, nonsense mutations, canonical splicing mutations, in-frame indels of ≥5 amino acids, and large rearrangements; non-truncating-type mutations included missense mutations, in-frame indels of ≤4 amino acids, and non-canonical splicing mutations [10].

*PKD1* mutations were classified into mutation strength groups (MSGs), according to previously reported methods [10]. Truncating-type *PKD1* mutations were defined as MGS1. Non-truncating-type *PKD1* mutations were further divided into strongly predicted mutations (MGS2), and less strongly predicted mutations (MSG3), by using similar criteria, such as substitution scores [5].

### Classification of *PKD1* mutation bisection positions and domain positions

Two bisection positions were selected at nucleotide #7978, as an equal group of patients in the present study (5′-segment: #1–#7978; 3′-segment: #7979–#12,912), and at nucleotide #6456 as a cDNA midpoint (5′-segment: nucleotide positions #1–#6456; 3′-segment: nucleotide positions #6457–#12,912).

*PKD1* mutation positions were classified into three domains, using nucleotide positions: the GPS-upstream domain, #1–#9183; transmembrane domain, #9223–#12,318 and the CTT domain, #12,319–#12,909.

### Statistical analyses

Parametric variables are expressed as mean ± standard deviation or standard error. Effects of covariates (genotypes, mutation types, and mutation positions) on the cumulative probability of renal and general survival were analyzed using the Kaplan–Meier method, and the univariate Cox’s proportional hazards model. For renal survival analysis, living subjects not undergoing RRT at the time of the study and dead subjects without RRT were considered as censored subjects, while those undergoing RRT were considered as non-censored subjects. In non-censored subjects, the survival year indicated the age at which the subject had started RRT. For general survival analysis, the survival year indicated the age at the inclusion in the study for living subjects (censored subjects), or the age of death for dead subjects (non-censored subjects).

The effects of covariates on continuous and categorical variables were examined using an analysis of variance, and Pearson’s Chi-squared test, respectively. The hazard ratio (HR) is shown, with a 95% confidence interval (CI).

All statistical analyses were performed using JMP^®^ ver. 10.0.0 Basic Analysis and Graphing (SAS Institute Inc., Cary, NC). All the tests were two-sided, and *P* < 0.05 was considered statistically significant.

## Results

### Distribution of pathogenic *PKD1* and *PKD2* mutations

Likely pathogenic ADPKD mutations were identified in 94 of 101 families (mutation detection rate, 93.1%), of which 82 families (81.2%) had *PKD1*, and 12 families (11.9%) had *PKD2* mutations. Truncating-type mutations were detected in 59.8% families with *PKD1,* and in 75.0% of families with *PKD2* mutations. Using MLPA, we detected large rearrangements in four patients, in whom NGS did not detect pathogenic mutations (Table 1). Sixty pathogenic mutations were newly described in 65 pedigrees, of which 57 were *PKD1*, and 8 *PKD2* mutations (Supplemental Table 1). These novel mutations have been described elsewhere [23].

**Table 1:** Distribution of pathogenic *PKD1* and *PKD2* mutations

Gene/mutation type		Pedigrees, *n* (%)	
*PKD1*	82 (81.2%)		
Truncating mutation		49 (59.8%)	
Frameshift			29
Nonsense			10
Splice (canonical)			6
Large rearrangements			4
Non-truncating mutation		33 (40.2%)	
Missense			26
Splice (non-canonical)			4
Inframe change (<4 amino acids)			3
*PKD2*	12 (11.9%)		
Truncating mutation		9 (75.0%)	
Frameshift			4
Nonsense			3
Splice			2
Non-truncating mutation		3 (25.0%)	
Missense			3
Mutation negative pedigrees	7 (6.9%)		

### Demographic characteristics of subjects included in survival analyses

Demographic data for 301 non-ADPKD family members, 338 patients with *PKD1* mutations, and 72 patients with *PKD2* mutations are presented in Table 2.

**Table 2:** Demographic characteristics of subjects used for survival analyses

	Non-ADPKD family member	Patients with *PKD1* mutation	Patients with *PKD2* mutation	Pearson’s Chi-square test	
				Three groups	PKD1 vs PKD2
Number of pedigrees	71	82	12	–	–
Subjects used for survival analyses	301	338	72	–	–
Men/women	151/150	159/179	35/37	0.7325	0.8086
Alive/dead	238/63	196/142	40/32	*P* < 0.0001	0.7045
RRT (−)/(+)	300/1	214/124	64/8	*P* < 0.0001	*P* < 0.0001
Paternal/maternal/unknown	–	99/149/90	25/31/16	–	0.5948

*RRT* renal replacement therapy

Affected and unaffected members of families with ADPKD showed non-significant differences with respect to gender distribution (*P* = 0.7325). However, survival and renal survival differed significantly between these groups (*P* < 0.0001 for both; Table 2). Renal survival also differed between patients with *PKD1* and *PKD2* mutations (*P* < 0.0001, Table 2).

### Genic effect

**Fig. 1:**
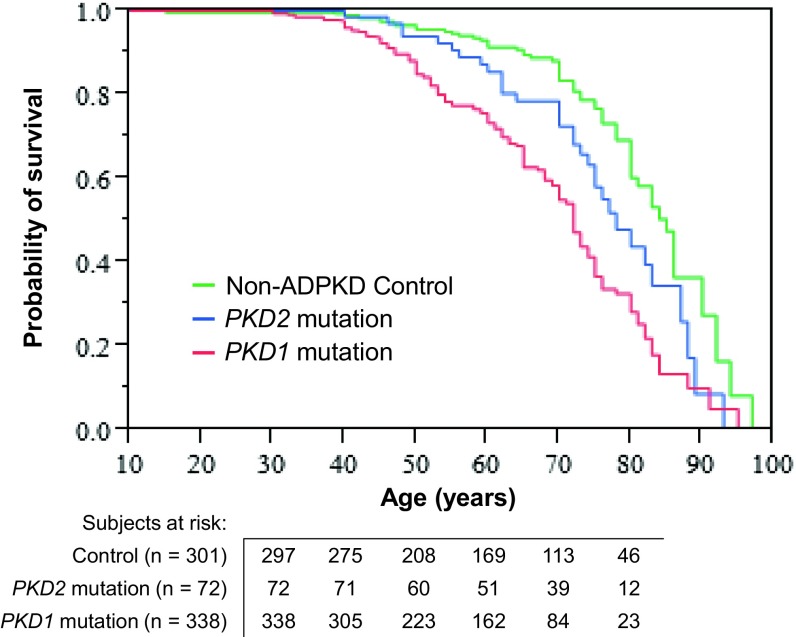
Kaplan–Meier analysis, showing survival curves of non-ADPKD family members and of patients with *PKD2* and *PKD1* mutations. Mean ± SE survival is significantly different among non-ADPKD family members (81.7 ± 1.2 years), patients with *PKD2* mutations (76.3 ± 1.9 years), and patients with *PKD1* mutations (69.7 ± 1.1 years) (log rank test, *P* < 0.0001). *PKD* genic mutations severely affect patient survival, as well as renal survival

Mean renal survival was shorter by 8.4 years in patients with *PKD1* mutations, than in those with *PKD2* mutations (*P* < 0.0001; Table 3; Supplemental Fig. 1). Life survival was also significantly different between non-PKD family members, and patients with *PKD1* and *PKD2* mutations (*P* < 0.0001; Supplemental Table 2; Fig. 1).

**Table 3:** *PKD* genic and *PKD1* allelic influences on renal survival

Genic and allelic variables	Subjects (*n*)	Renal survival (years) by Kaplan–Meier analysis			Cox’s proportional hazards analysis		
		Mean	SE	*P* value	Univariate HR	95% CI	*P* value
*PKD* genic influence							
*PKD1*	338	66.87	0.98	Log rank test, *P* < 0.0001 Wilcoxon test, *P* < 0.0001	6.8	3.54–15.18	<0.0001
*PKD2*	72	75.22	0.72		1 (referent)		
*PKD1* allelic influence							
*PKD1* mutation type							
Non-truncating	134	69.14	1.50	Log rank test, *P* = 0.0348 Wilcoxon test, *P* = 0.0595	1 (referent)		
Truncating	204	64.81	1.22		1.47	1.02–2.14	0.0365
*PKD1* mutation strength group (MSG)							
MSG 1	204	64.81	1.22	Log rank test, *P* = 0.1072 Wilcoxon test, *P* = 0.1174	1.50	0.95–2.47	0.0864
MSG 2	75	65.33	1.55		1.03	0.58–1.85	0.9131
MSG 3	59	69.82	2.06		1 (referent)		
*PKD1* mutation type and position							
All *PKD1* mutations							
5′-end position	174	67.49	1.28	Log rank test, *P* = 0.2263 Wilcoxon test, *P* = 0.2027	1 (referent)		
3′-end position	164	65.69	1.42		1.24	0.87–1.77	0.2346
Truncating-type *PKD1* mutations							
5′-end position	125	64.01	1.53	Log rank test, *P* = 0.4375 Wilcoxon test, *P* = 0.8920	1 (referent)		
3′-end position	79	65.30	1.94		0.84	0.52–1.32	0.4452
Non-truncating-type *PKD1* mutations							
5′-end position	49	71.29	1.54	Log rank test, *P* = 0.0014 Wilcoxon test, *P* = 0.0041	1 (referent)		
3′-end position	85	65.11	1.91		2.72	1.46–5.38	0.0013
Non-truncating-type *PKD1* mutations							
GPS-upstream domain	56	70.17	1.49	Log rank test, *P* = 0.0001 Wilcoxon test, *P* < 0.0001	1 (referent)		
Transmembrane domain	66	67.01	2.19		1.94	1.05–3.68	0.0354
CCT domain	12	50.07	1.67		6.61	2.32–16.53	0.0010

5′-region position: nucleotide position #1–#7978; 3′-region position: nucleotide position #7979–#12,912. GPS-upstream domain: nucleotide position #1–#9183; Transmembrane domain: nucleotide position #9223–#12,318; CCT domain: nucleotide position #12,319–#12,909

*GPS* G-protein-coupled receptor proteolytic site, *CCT* cytoplasmic C-terminal tail

### Allelic influences of *PKD1* mutations on renal survival

#### Influences of *PKD1* mutation types and MSGs

**Fig. 2:**
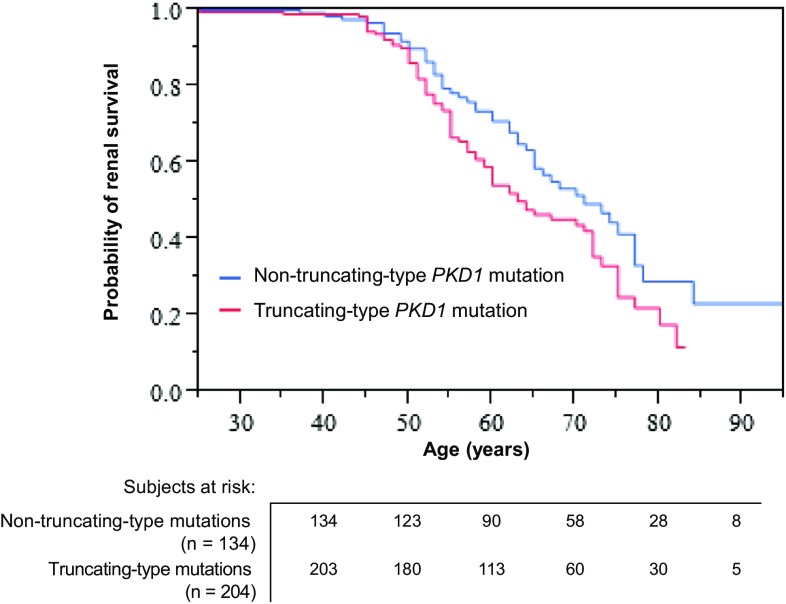
Renal survival plots of carriers of *PKD1* non-truncating- and truncating-type mutations. *PKD1* mutation types affect renal survival (log rank test, *P* = 0.0348). The mean age difference is 4.3 years. The different renal survival is explained by the complete or incomplete inactivation of *PKD1*

Carriers of truncating-type *PKD1* mutations displayed shorter renal survival than carriers of non-truncating-type *PKD1* mutations, according to the Kaplan–Meier analysis (log rank test, *P* = 0.0348; Table 3; Fig. 2), and Cox’s proportional hazard analysis (*P* = 0.0365; Table 3).

The influence of *PKD1* MSGs on renal survival was not significant (Table 3; Supplemental Fig. 2). However, renal survival times tended to be longer in patients in the MSG3 group than in those in the MSG1 group (log rank test, *P* = 0.0605; Wilcoxon test, *P* = 0.0437, according to the Kaplan–Meier analysis, and *P* = 0.0573, according to Cox’s proportional hazard analysis).

#### Influences of the positions of *PKD1* mutations, according to mutation types

The influences of the positions of *PKD1* mutations were analyzed separately for truncating- and non-truncating-type mutations, because their effects on the structure of PC1 were assumed to differ, according to mutation types. Truncating-type mutations affect all downstream amino acid sequences from the mutated position, whereas non-truncating-type mutations are expected to only weakly or locally affect downstream amino acid sequences, although they may alter the folding and localization of the protein [17].

Bisection positions were selected at nucleotide #7978 (Table 3) and #6456 (Supplemental Table 3), as explained in the “Materials and methods”. The effects of the bisection positions on renal survival were examined in three groups, namely, in the truncating-plus non-truncating-type (all), truncating-type, and non-truncating-type mutation groups.

**Fig. 3:**
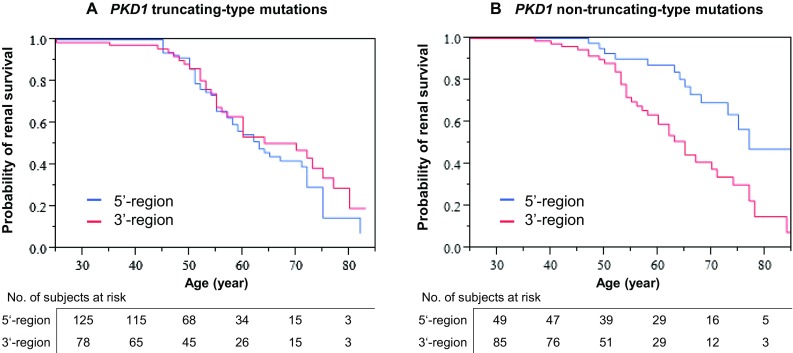
Position (5′- versus 3′-region, divided at nucleotide #7978) of **a** truncating- and **b** non-truncating-type mutations, showing different effects on renal survival. **b** The position of non-truncating-type mutations has a significant influence (log rank test, *P* = 0.0014), whereas **a** that of truncating-type mutations does not have a significant influence (log rank test, *P* = 0.4375). This difference might be due to the uniform inactivation of the CTT domain, irrespective of the position of the mutation in **a**, compared to the variability of CTT inactivation in **b**, based on the location of mutation sites. See Fig. 4

In the truncating-type and truncating- plus non-truncating-type mutation groups, renal survival did not differ between patients with mutations in the 5′- and 3′-regions, divided at nucleotide #7978. In contrast, in the non-truncating-type mutation group, renal survival was shorter in carriers of mutations in the 3′-region than in carriers of mutations in the 5′-region (log rank test, *P* = 0.0014; Table 3; Fig. 3). Qualitatively similar differences were also confirmed between the 3′- and 5′-positions which were divided at nucleotide #6456 (Supplemental Table 3).

**Fig. 4:**
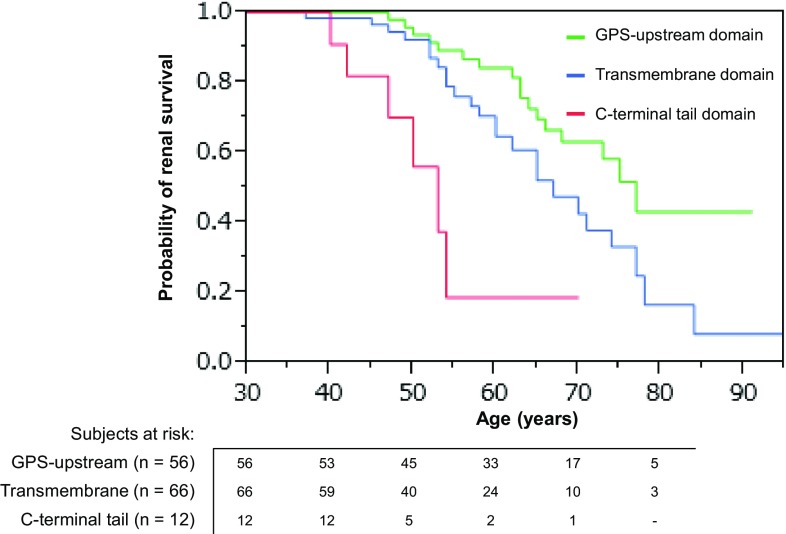
Kaplan–Meier analysis, showing renal survival curves of patients with *PKD1* non-truncating-type mutations in the GPS-upstream domain, transmembrane domain, and CTT domain. Mean ± SE renal survival is significantly different among patients with non-truncating-type mutations in the GPS-upstream domain (70.2 ± 1.5 years), in the transmembrane domain (67.0 ± 2.2 years), and in the CTT domain (50.1 ± 1.7 years) (log rank test, *P* < 0.0001). This figure indicates a possible crucial role of the CTT domain in PC1 function

The influence of non-truncating-type mutation positions on renal survival was further examined by dividing the position of the mutations into the GPS-upstream domain, the transmembrane domain, and the CTT domain. Renal survival was longer in the above order (log rank test, *P* = 0.0001; Table 3; Fig. 4), and Cox’s hazard analysis confirmed a significant difference between each of the domains (Table 3). In truncating-type mutations, however, no significant difference was observed on renal survival among three domains (log rank test, *P* = 0.6551 according to the Kaplan–Meier analysis, and *P* = 0.6447 according to Cox’s proportional hazards analysis).

### Allelic influences of *PKD1* mutations on survival

*PKD1* mutation types did not have a significant influence on survival (Supplemental Table 2). Carriers of 5′-end mutations showed longer survival times than those of 3′-end mutations, when the groups were divided at nucleotide #6456, but the difference was not significant when divided at nucleotide #7978. In non-truncating-type mutation carriers, survival was shorter in 3′-end mutation carriers than in 5′-end mutation carriers (log rank test, *P* = 0.0053; Supplemental Table 2). Survival was also significantly different among carriers of *PKD1* non-truncating-type mutations in the GPS-upstream domain, the transmembrane domain, and the CTT domain, and it became shorter in this order (log rank test, *P* = 0.0036; Supplemental Table 2).

### Effects of sex and parental origin

Women showed a trend of longer renal survival than men; however, the difference was not statistically significant (Table 4).

**Table 4:** Effects of gender and parental origin on renal survival

Variables	Subjects (*n*)	Survival (years) by Kaplan–Meier analysis			Cox’s proportional hazards analysis		
		Mean	SE	*P* value	Univariate HR	95% CI	*P* value
Gender							
All *PKD*							
Female	216	70.25	1.10	Log rank test, *P* = 0.1729 Wilcoxon test, *P* = 0.2022	1 (referent)		
Male	194	68.68	1.32		1.26	0.90–1.78	0.1800
*PKD1*							
Female	179	67.67	1.28	Log rank test, *P* = 0.1444 Wilcoxon test, *P* = 0.1866	1 (referent)		
Male	159	65.32	1.41		1.29	0.91–1.84	0.1524
*PKD2*							
Female	37	74.96	1.05	Log rank test, *P* = 0.4677 Wilcoxon test, *P* = 0.5837	1 (referent)		
Male	35	67.64	0.50		0.56	0.08–2.44	0.4592
Parental origin							
All *PKD*							
Maternal	180	69.80	1.25	Log rank test, *P* = 0.1743 Wilcoxon test, *P* = 0.0565	1 (referent)		
Paternal	124	65.62	1.36		1.31	0.88–1.96	0.1831
*PKD1*							
Maternal	149	67.93	1.43	Log rank test, *P* = 0.0039 Wilcoxon test, *P* = 0.0104	1 (referent)		
Paternal	99	61.53	1.46		1.84	1.20–2.83	0.0054
*PKD2*							
Maternal	31	68.77	0.95	Log rank test, *P* = 0.1444 Wilcoxon test, *P* = 0.1222	1 (referent)		
Paternal	25	76.11	1.23		0.33	0.05–1.42	0.1417

Parental origin could not be determined in 106 patients

*ESRD* end-stage renal disease

The parental origin of mutations was identified in 304 patients. Patients inheriting *PKD1* mutations from their fathers (paternal origin) showed shorter renal survival than those inheriting *PKD1* mutations from their mothers (maternal origin) (log rank test, *P* = 0.0039; Table 4).

## Discussion

The identification of pathogenic mutations by using a combination of NGS, LR-PCR, MLPA, and an analysis software package provided an overall *PKD* mutation detection rate of 93.1% (94/101) [23]. This detection rate was similar (84.5–93.8%) to that reported in recent large-scale *PKD* studies [5, 7, 8, 10, 12].

*PKD1* and *PKD2* pathogenic mutations accounted for 81.2 and 11.9% of pedigrees, respectively (Table 1). These percentages of patients with *PKD1* and *PKD2* mutations are not significantly different from those reported in previous studies [5, 7, 8, 10, 12]. The ratio of patients with *PKD1* to *PKD2* mutations found by recent gene analyses seems approximately uniform, despite different ethnic groups, when the study avoids the preferential inclusion or exclusion of patients receiving RRT. In patients with *PKD1* mutations, 59.8% of pedigrees exhibited truncating-type mutations (Table 1). This percentage was slightly lower than that reported in previous studies (65.1–69.9%), but the difference was not statistically significant [5, 7, 10].

Renal survival was reported to be longer by approximately 20 years in patients with *PKD2* mutations than in patients with *PKD1* mutations [8, 11, 26]. In this study, the mean and median ages of patients with *PKD1* mutations, at the onset of ESRD, were 66.9 and 67 years (95% CI 63–72), respectively. The median ages at the onset of ESRD in the Catalan [11], European [26], and Genkyst [8] studies were 53.4, 54.3, and 58.1 years, respectively. The mean renal survival in patients with *PKD2* mutations was 75.2 years in the present study (Table 3), which was within the reported range (69.1–79.7 years) [8, 11, 26].

The age at the onset of ESRD increased in later studies. An improved renal prognosis after approximately 8 years was observed in patients with ADPKD [27]. According to the annual report of the Japanese Society for Dialysis Therapy, the mean age of patients with ADPKD at the onset of dialysis therapy was 54.8 years in 1987, and increased to 63.1 years in 2014 [28]. The delayed onset of dialysis therapy was generally recognized in other chronic kidney diseases. The shorter interval, at the onset of ESRD, between patients with *PKD1* and *PKD2* mutations, in the present study, might be explained by the preferential improvement of the renal prognosis in patients with worse kidney function, such as patients with *PKD1*. In addition, the 5–10% lower fraction of truncating-type mutations in these patients (Table 1) might explain, in part, their longer renal survival. The factors mentioned above (renal survival was improved in recent reports and relatively small percentage of patients with truncating-type mutation) might partly explain the longer renal survival in patients with *PKD1* mutation of this study than those reported previously [8, 11, 26]. Other factors might be possible and large number study is needed.

Most studies involving a relatively large population have reported improved renal survival in women compared to that in men [4, 8, 10, 29]; however, some studies did not report this sex-related difference in patients with *PKD1* mutations [13, 26]. The tendency of favorable renal survival in women with *PKD1* mutations was recognized, but the difference was not significant, probably because of the small number of patients included in this study.

A previous study has shown that the parental inheritance of *PKD1* or *PKD2* mutations did not affect the survival to death, or ESRD development, in patients with ADPKD [26]. In contrast, the results of the present study showed poorer renal survival in patients with *PKD1* mutations of paternal origin, than in those with *PKD1* mutations of maternal origin (mean survival, 61.5 versus 67.9 years; *P* = 0.0039; Table 4). Analytical endpoint was survival to death or ESRD in previous study [26] and that was survival to ESRD alone in this study. The different end point utilized in these studies might explain the different result in survival concerning parental inheritance.

Recent studies have reported severely impaired renal survival and poor eGFR in patients with *PKD1* truncating-type mutations, compared with those in patients with *PKD1* non-truncating-type mutations [8, 10, 12]. The htTKV did not differ between patients with truncating- and non-truncating-type mutations, but did differ between patients in the MSG1 and MSG3 groups [10]. The present study confirmed the severely impaired renal survival in carriers of *PKD1* truncating-type mutations, compared with that in carriers of *PKD1* non-truncating-type mutations (Table 3; Fig. 2). These *PKD1* allelic influences suggest that the complete inactivation of PC1 results in a severe renal phenotype [8, 10].

A previous study has reported that renal survival was shorter in patients with *PKD1* mutations in the 5′-region than in patients with *PKD1* mutations in the 3′-region (53 and 56 years, respectively; *P* = 0.025) [13]. However, renal survival plots comparing *PKD1* mutations in the 5′- and 3′-regions did not show any significant difference (*P* = 0.69) in another study [8], and in the present study (log rank test, *P* = 0.2263; Table 3). This difference might be partly explained by improvements in both molecular analyses and the scoring of missense mutations in the last 10 years, which resulted in identification of a significant number of mild or “hypomorphic” alleles [8]. Our observations support this report.

The influence of the position of the mutation on renal survival was strikingly different between patients with truncating- and non-truncating-type *PKD1* mutations (Table 3; Fig. 3). This influence was significant based on Cox’s hazard analysis in patients with non-truncating-type *PKD1* mutations (*P* = 0.0013; Table 3), but was not significant in patients with truncating-type *PKD1* mutations (*P* = 0.4452; Table 3). The differences in the renal phenotypes between carriers of 3′-end non-truncating-type mutations and carriers of 5′-end non-truncating-type mutations was confirmed by the differences in renal survival among carriers harboring mutations in three distinct domains. The renal prognosis for carriers of non-truncating-type *PKD1* mutations became more severe in the following order: GPS-upstream, transmembrane, and CTT domain mutations (Table 3; Fig. 4; Supplemental Fig. 3). These findings might be in accordance with the essential role of PC1–PC2 complex formation through the interaction of their CTT domains for mechanosensory PC function in the primary cilium [16–18]. Topological relationship among three domains, equal group mutation position around median nucleotide #7978 and cDNA midpoint (nucleotide #6456) is illustrated in Supplemental Fig. 3.

Dissimilar *PKD1* allelic influences between truncating and non-truncating mutations were explained by the complete inactivation of PC1 in truncating-type mutations [8, 10]. The complete inactivation of PC1 may not be affected by positional differences (3′ vs 5′), with respect to the expressed phenotype. In non-truncating-type mutations, on the other hand, the variability in renal survival due to positional differences may be recognized, due to the incomplete inactivation of PC1 (Table 3; Fig. 3).

However, the results of the present study provide additional insights into the dissimilar allelic influence on the renal phenotype. Irrespective of the position of the mutation (in the 3′- or 5′-region), the inactivation of the CTT domain is expected in truncating-type mutations. Hence, differences in renal survival were not observed between patients with mutations in the 3′- or the 5′-region. In addition, the inactivation of the CTT domain is heterogeneous, based on the positions of the mutations, in non-truncating-type mutations. Hence, a milder phenotype is expressed in non-truncating-type mutation carriers than in truncating-type mutation carriers, due to the reduced inactivation of the CTT domain in non-truncating-type mutations.

The influence of mutated loci on renal survival has been rarely reported in patients with ADPKD. A possible correlation between the mutated loci and the disease phenotype has been reviewed in patients with cystic fibrosis [30]. However, additional genotype and phenotype correlation studies are needed to explore the integrated relationships between mutation types and positions in patients with ADPKD [31–33].

A limitation of this study is the inclusion of a relatively small number of patients, compared with that in large-scale *PKD* studies [8, 10]. However, the present study confirmed the significant allelic influence of *PKD1* mutation types on renal survival, which is consistent with that reported in previous large-scale studies [8, 10]. In addition, our results suggested a potentially crucial role for the CTT domain in renal prognosis, which requires a more comprehensive elucidation in future larger scale studies.

## Electronic supplementary material

Below is the link to the electronic supplementary material.

Supplementary material 1 (DOCX 36 kb)

Supplementary material 2 (PPTX 104 kb)

Supplementary material 3 (DOCX 15 kb)
