# Supplementary material for: A potentially crucial role of the PKD1 C-terminal tail in renal prognosis
Source: Clin Exp Nephrol. 2017 Oct 5;22(2):395–404. doi: 10.1007/s10157-017-1477-7 (PMC5838153; doi:10.1007/s10157-017-1477-7)
Supplement: Supplementary file 1 — Supplementary material 1 (DOCX 36 kb) [file 10157_2017_1477_MOESM1_ESM.docx]

| **Supplemental Table 1. Characteristics of pathogenic *PKD* mutations** | | | |
| --- | --- | --- | --- |
|  | *PKD1* | *PKD2* | Total |
| Pedigrees with pathogenic mutations | 82 | 12 | 94 |
| Pedigrees with novel pathogenic mutations | 56 | 8 | 64 |
| (Novel pathogenic mutations) | (52) | (8) | (60) |
| Different pathogenic mutations | 73 | 10 | 83 |
| Identical pathogenic mutations | 7 | 1 | 8 |
| Pathogenic mutations in a single pedigree | 66 | 9 | 75 |
| Pedigrees with identical pathogenic mutations | 16 | 3 | 19 |

Novel pathogenic mutations of *PKD1* included 4 nonsense mutations, 19 (1) frameshift mutations, 4 large rearrangements, one (1) in-frame change < 3amino acid, 6 atypical splicing and 18 (2) missense mutations. Novel pathogenic mutations of *PKD2* included 2 nonsense mutations, 4 frameshift mutations and 2 atypical splicing. Numbers in parentheses are numbers of identical mutations. [from reference 23]

| **Supplemental Table 2. *PKD* genic and *PKD1* allelic influences on life survival** | | | | | | | |
| --- | --- | --- | --- | --- | --- | --- | --- |
| Genotype and allelic variables | | | | Subjects | Survival (years) | | |
|  |  |  |  | (n) | Mean | SE | P value |
| *PKD* genic influence | | | | |  |  |  |
|  |  |  | Non-*PKD* family member | 301 | 81.68 | 1.19 | Log rank test, P < 0.0001 Wilcoxon test, P < 0.0001 |
|  |  |  | *PKD1* | 338 | 69.70 | 1.09 |  |
|  |  |  | *PKD2* | 72 | 76.34 | 1.86 |  |
| *PKD1* allelic influence | | | | |  |  |  |
|  | All mutation types | | |  |  |  |  |
|  |  | Truncating vs non-truncating | | |  |  |  |
|  |  |  | Non-truncating | 134 | 70.60 | 1.66 | Log rank test, P = 0.3779 Wilcoxon test, P = 0.5585 |
|  |  |  | Truncating | 204 | 67.66 | 1.15 |  |
|  |  | Mutation strength group | | |  |  |  |
|  |  |  | MSG 1 | 204 | 67.66 | 1.15 | Log rank test, P = 0.5999 Wilcoxon test, P = 0.7082 |
|  |  |  | MSG 2 | 75 | 70.03 | 2.30 |  |
|  |  |  | MSG 3 | 59 | 71.20 | 2.27 |  |
|  |  | Mutation position (Midpoint: #6,456) | | |  |  |  |
|  |  |  | 5ʹ-end position | 132 | 72.31 | 1.70 | Log rank test, P = 0.0453 Wilcoxon test, P = 0.0360 |
|  |  |  | 3ʹ-end position | 206 | 68.26 | 1.42 |  |
|  |  | Mutation position (Midpoint: #7,978) | | |  |  |  |
|  |  |  | 5ʹ-end position | 174 | 70.45 | 1.43 | Log rank test, P = 0.4482 Wilcoxon test, P = 0.1169 |
|  |  |  | 3ʹ-end position | 164 | 69.00 | 1.66 |  |
|  | Truncating- or non-truncating-type | | | | |  |  |
|  |  | Truncating-type mutations (Midpoint: #7,978) | | | | |  |
|  |  |  | 5ʹ-end position | 125 | 66.55 | 1.44 | Log rank test, P = 0.1988 Wilcoxon test, P = 0.6733 |
|  |  |  | 3ʹ-end position | 79 | 69.14 | 1.91 |  |
|  |  | Non-truncating-type mutations (Midpoint: #7,978) | | | | |  |
|  |  |  | 5ʹ-end position | 49 | 76.82 | 2.28 | Log rank test, P = 0.0053 Wilcoxon test, P = 0.0012 |
|  |  |  | 3ʹ-end position | 85 | 66.70 | 2.13 |  |
|  |  | Non-truncating-type mutations | | | | |  |
|  |  |  | GPS-upstream domain | 56 | 76.07 | 2.11 | Log rank test, P = 0.0036 Wilcoxon test, P = 0.0009 |
|  |  |  | Transmembrane domain | 66 | 67.36 | 2.51 |  |
|  |  |  | CTT domain | 12 | 58.41 | 3.49 |  |
| Nucleotide positions #6,456 and #7,978 were used as a structural midpoint and a distribution midpoint, respectively. GPS-upstream domain: nucleotide position #1-#9,183; Transmembrane domain: nucleotide position #9,223-#12,318; CTT domain: nucleotide position #12,319-#12,909. GPS, G protein-coupled receptor proteolytic site. CTT, Cytoplasmic C-terminal tail. | | | | | | | |

| **Supplemental Table 3. Influence of *PKD1* mutation types and bisection positions (midpoint nucleotide #6,456) on renal survival** | | | | | | | | | | | |
| --- | --- | --- | --- | --- | --- | --- | --- | --- | --- | --- | --- |
| Allelic variables | | | Subjects |  | Survival (years) by Kaplan-Meier analysis | | |  | Cox's proportional hazards analysis | | |
|  |  |  | (n) |  | Mean | SE | P value |  | Univariate HR | 95% CI | P value |
|  | All *PKD1* mutations | |  |  |  |  |  |  |  |  |  |
|  |  | 5ʹ-end position | 132 |  | 68.87 | 1.47 | Log rank test, P = 0.0293 Wilcoxon test, P = 0.0473 |  | 1 (referent) |  |  |
|  |  | 3ʹ-end position | 206 |  | 65.20 | 1.25 |  |  | 1.50 | 1.04-2.20 | 0.0301 |
|  | Truncating-type *PKD1* mutations | |  |  |  |  |  |  |  |  |  |
|  |  | 5ʹ-end position | 99 |  | 65.84 | 1.81 | Log rank test, P = 0.4005 Wilcoxon test, P = 0.2470 |  | 1 (referent) |  |  |
|  |  | 3ʹ-end position | 105 |  | 63.52 | 1.63 |  |  | 1.21 | 0.77-1.92 | 0.4094 |
|  | Non-truncating-type *PKD1* mutations | |  |  |  |  |  |  |  |  |  |
|  |  | 5ʹ-end position | 33 |  | 72.00 | 1.80 | Log rank test, P = 0.0040 Wilcoxon test, P = 0.0133 |  | 1 (referent) |  |  |
|  |  | 3ʹ-end position | 101 |  | 66.18 | 1.77 |  |  | 2.81 | 1.40-6.25 | 0.0028 |
| 5ʹ- and 3ʹ-regions are divided at nucleotide position #6,456. | | | | | | | | | | | |
