# Supplementary material for: A potentially crucial role of the PKD1 C-terminal tail in renal prognosis
Source: Clin Exp Nephrol. 2017 Oct 5;22(2):395–404. doi: 10.1007/s10157-017-1477-7 (PMC5838153; doi:10.1007/s10157-017-1477-7)
Supplement: Supplementary file 2 — Supplementary material 2 (PPTX 104 kb) [file 10157_2017_1477_MOESM2_ESM.pptx]

## Slide 1
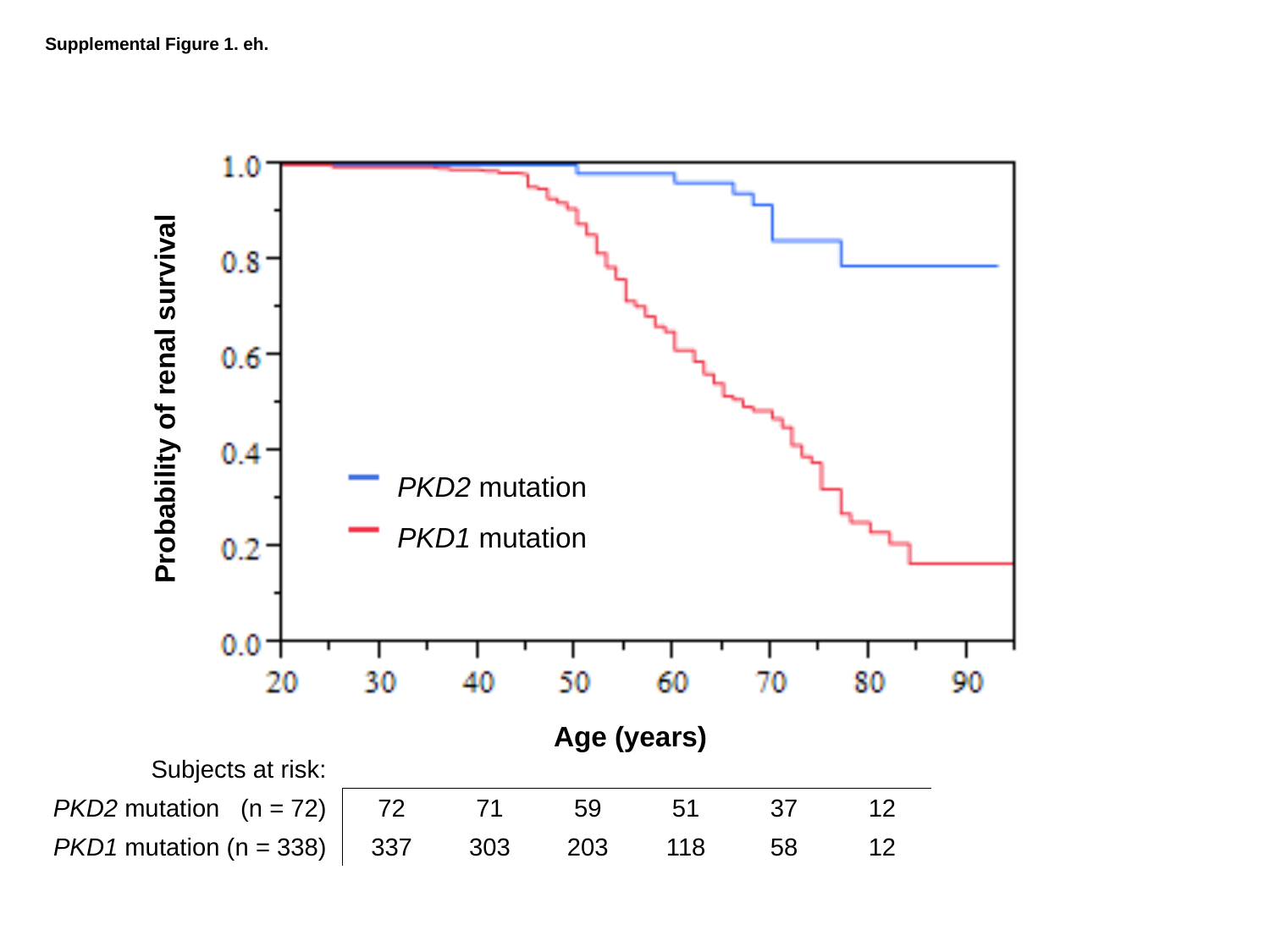

Supplemental Figure 1. eh.
Probability of renal survival
PKD2 mutation
PKD1 mutation
Age (years)
| Subjects at risk: | | | | | | | |
| --- | --- | --- | --- | --- | --- | --- | --- |
| PKD2 mutation (n = 72) | | 72 | 71 | 59 | 51 | 37 | 12 |
| PKD1 mutation (n = 338) | | 337 | 303 | 203 | 118 | 58 | 12 |

## Slide 2
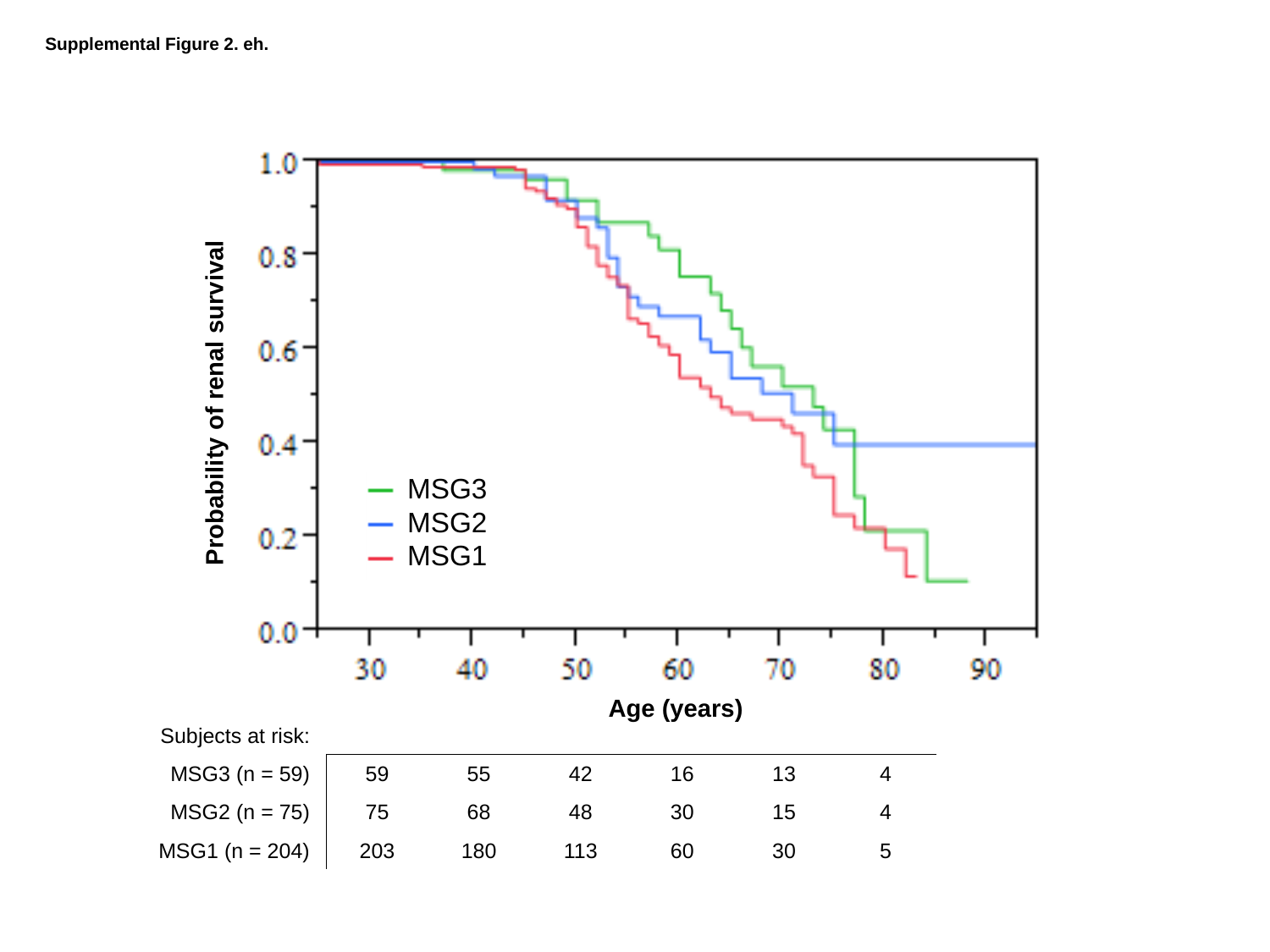

Supplemental Figure 2. eh.
Probability of renal survival
MSG3
MSG2
MSG1
Age (years)
| Subjects at risk: | | | | | | | |
| --- | --- | --- | --- | --- | --- | --- | --- |
| MSG3 (n = 59) | | 59 | 55 | 42 | 16 | 13 | 4 |
| MSG2 (n = 75) | | 75 | 68 | 48 | 30 | 15 | 4 |
| MSG1 (n = 204) | | 203 | 180 | 113 | 60 | 30 | 5 |

## Slide 3
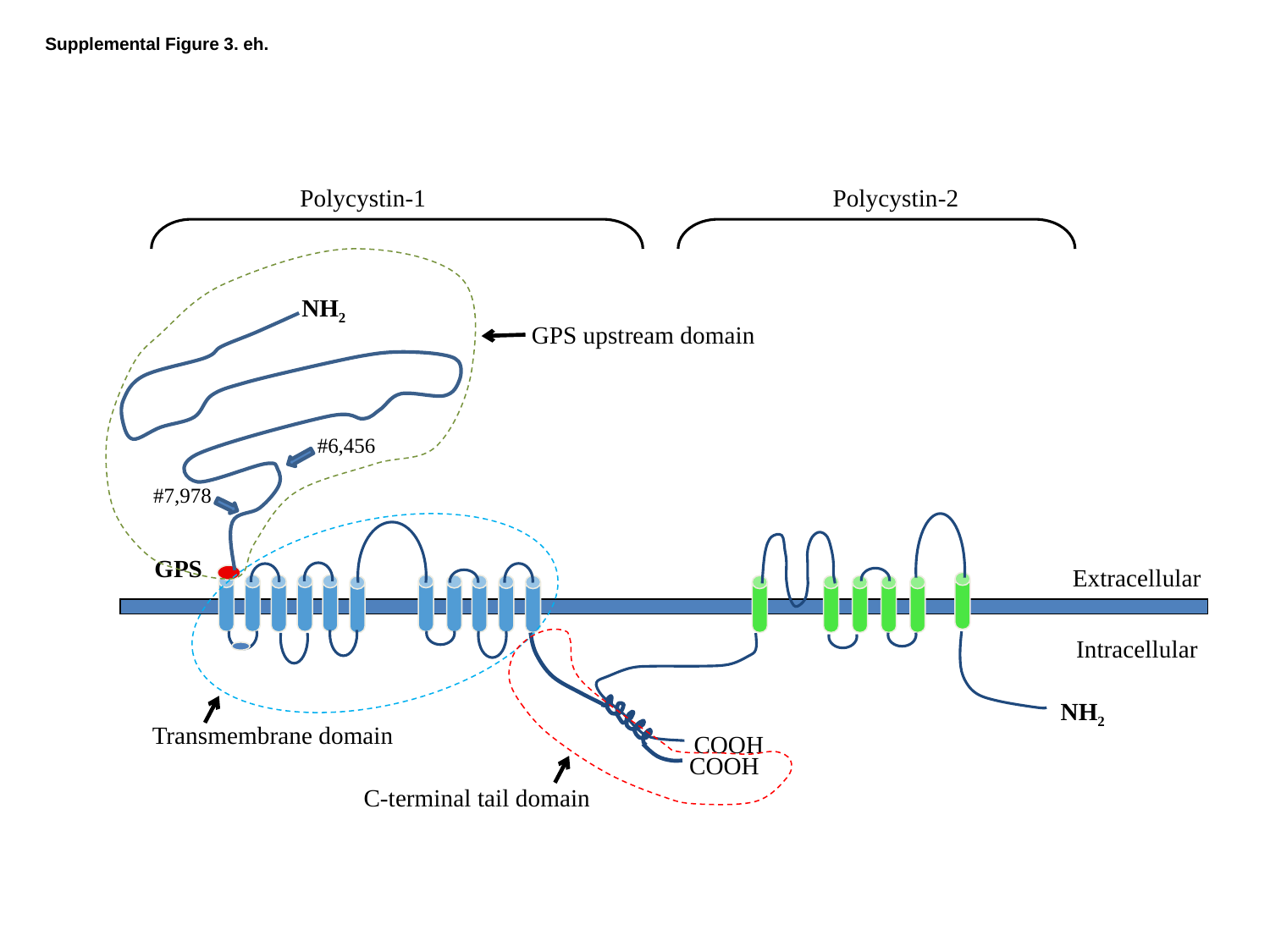

Supplemental Figure 3. eh.
Polycystin-1
Polycystin-2
NH2
GPS upstream domain
#6,456
#7,978
GPS
Extracellular
Intracellular
NH2
Transmembrane domain
COOH
COOH
C-terminal tail domain
