# Supplementary material for: A potentially crucial role of the PKD1 C-terminal tail in renal prognosis
Source: Clin Exp Nephrol. 2017 Oct 5;22(2):395–404. doi: 10.1007/s10157-017-1477-7 (PMC5838153; doi:10.1007/s10157-017-1477-7)
Supplement: Supplementary file 3 — Supplementary material 3 (DOCX 15 kb) [file 10157_2017_1477_MOESM3_ESM.docx]

Supplemental Figure 1

Renal survival plots of patients with *PKD1* and *PKD2* mutations.

The renal survival curves are significantly different between the two groups (log rank test, *P* < 0.0001).

Supplemental Figure 2

Renal survival plots of *PKD1* mutations, according to MSGs.

The renal survival curves are not significantly different between patients in the three MSGs (log rank test, *P* = 0.1072). However, the renal survival curves of patients in the MSG3 group tend to be associated with a better outcome, than those of patients in the MSG1 group (log rank, *P* = 0.0605; Wilcoxon test, *P* = 0.0437).

Supplemental Figure 3

Topology of GPS-upstream domain (#1-#9,183), transmembrane domain (#9,223-#12,318), C-terminal tail domain (#12,319–#12,909), equal group mutation position around median: nucleotide #7,978 and midpoint (#6,456) of polycystin-1 cDNA are illustrated each in a proposed structural model of polycystin-1 and polycystin-2.
